# Fibrosis‐Driven Surgical Risk After Thyroid Nodule Ablation: Quantitative Clinicopathological Determinants of Complications in Post‐Ablative Thyroidectomy—A Retrospective Cohort Study

**DOI:** 10.1002/wjs.70300

**Published:** 2026-03-08

**Authors:** Daqi Zhang, Alvino Boero, Giacomo Gazzano, Andrea Satta, Laura Fugazzola, Carla Colombo, Francesco Brucchi, Gianlorenzo Dionigi

**Affiliations:** ^1^ Division of Thyroid Surgery China‐Japan Union Hospital of Jilin University Jilin Provincial Key Laboratory of Thyroid Disease Jilin Provincial Precision Medicine Laboratory of Molecular Biology and Translational Medicine on Differentiated Thyroid Carcinoma Changchun Jilin China; ^2^ Division of General Surgery Istituto di Ricovero e Cura a Carattere Scientifico (IRCCS) Istituto Auxologico Italiano Milan Italy; ^3^ Department of Pathophysiology and Transplantation University of Milan Milan Italy; ^4^ Pathology Unit IRCCS Istituto Auxologico Italiano Milan Italy; ^5^ Department of Endocrinology ASST Santi Paolo e Carlo Milan Italy; ^6^ Department of Health Sciences University of Milan Milan Italy; ^7^ Division of Endocrinology Istituto di Ricovero e Cura a Carattere Scientifico (IRCCS) Istituto Auxologico Italiano Milan Italy

**Keywords:** fibrosis and necrosis, histopathology, hypoparathyroidism, radiofrequency ablation, recurrent laryngeal nerve, surgical complications, thermal ablation, thyroid nodules, thyroidectomy, tissue remodeling

## Abstract

**Background:**

Thermal ablation is increasingly used for selected benign and low‐risk thyroid nodules, yet some patients still require thyroidectomy for regrowth, persistent symptoms, or new oncologic concern. The surgical and pathological impact of ablation‐induced remodeling remains incompletely defined. We aimed to characterize postablation thyroidectomy outcomes and identify histological correlates of perioperative morbidity.

**Methods:**

We conducted a single‐center retrospective cohort study of patients undergoing thyroidectomy after radiofrequency or ethanol ablation (2021–2025). Clinical and ablation‐related variables were collected, and intraoperative neuromonitoring was routinely used. Primary outcomes were recurrent laryngeal nerve (RLN) palsy, reoperative hematoma, and hypoparathyroidism. Surgical specimens underwent blinded dual‐pathologist assessment with semiquantitative scoring of sclerosis, necrosis, and residual viability, from which a maturation index was derived. Associations with complications were analyzed using nonparametric methods. Outcomes were descriptively compared with a contemporaneous nonablated cohort for contextual purposes.

**Results:**

Thirty‐one patients were included. Postoperative complications occurred in 22.6% of ablated cases. Histological analysis demonstrated moderate sclerosis (19.2%), necrosis (14.6%), and high residual viability (66.1%), with frequent pericapsular inflammatory changes and preserved capsule integrity. Sclerosis was the only parameter significantly associated with postoperative complications (30.0% vs. 16.9% and *p* = 0.008), whereas nodule size, ablation‐to‐surgery interval, and incidental carcinoma were not predictive. The maturation index increased with time after ablation but did not discriminate complication risk. Exploratory fibrosis‐weighted metrics suggested potential risk thresholds, although these findings remain hypothesis‐generating given the limited sample size.

**Conclusions:**

Thyroidectomy after prior ablation is feasible in experienced centers but may be technically demanding and associated with modestly increased procedural complexity. Mature sclerosis represents the principal histological correlate of perioperative morbidity, linking fibrotic remodeling to operative risk. These findings support centralization of postablation thyroid surgery in high‐volume units with routine neuromonitoring and specialized pathology and highlight the need for larger prospective studies to validate fibrosis‐based risk stratification tools.

## Graphical abstract

Thermal ablation is increasingly used for selected benign and low‐risk thyroid nodules, yet some patients still require thyroidectomy for regrowth, persistent symptoms, or new oncologic concern. The surgical and pathological impact of ablation‐induced remodeling remains incompletely defined. We aimed to characterize postablation thyroidectomy outcomes and identify histological correlates of perioperative morbidity. We conducted a single‐center retrospective cohort study of patients undergoing thyroidectomy after radiofrequency or ethanol ablation (2021–2025).

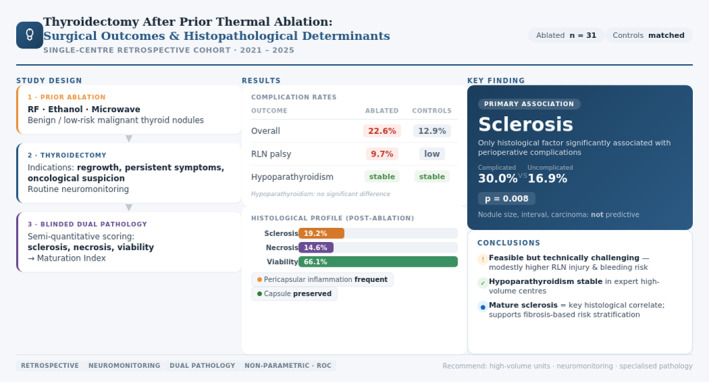

## 1 Introduction

Radiofrequency ablation (RFA) and other thermal techniques have become minimally invasive alternatives to surgery for selected thyroid nodules, including benign lesions, autonomously functioning nodules, and carefully chosen low‐risk papillary microcarcinomas [1, 2, 3, 4]. These modalities typically achieve 50%–80% volume reduction, with marked improvement in compressive and cosmetic symptoms and satisfactory local control in most low‐risk malignant nodules [5, 6, 7].

Despite these benefits, a proportion of patients experience regrowth or suboptimal local control, with recurrent compressive symptoms, persistent neck deformity, or new suspicious features requiring further evaluation and definitive treatment [8, 9, 10]. Multicenter series indicate that retreatment is not uncommon and that thyroidectomy represents a significant share of these procedures, occurring in roughly one in 10 patients initially treated with thermal ablation [11, 12]. This raises important questions about the technical and diagnostic impact of prior ablation on subsequent thyroidectomy [13, 14].

Thermal ablation induces characteristic tissue remodeling—hyalinization, coagulative necrosis, chronic inflammation, fibrosis, and perithyroidal adhesions—which can distort anatomical planes and involve critical central neck structures [15, 16, 17, 18, 19]. These changes may increase thyroidectomy difficulty, prolong operative time, and elevate the risk of RLN injury, hypoparathyroidism, and incidental parathyroidectomy compared with primary surgery [13, 14]. Macroscopic adhesion scores and thyroidectomy difficulty scales likewise indicate more severe adhesions in postablation procedures, although evidence remains limited and based on small cohorts [15, 16, 17, 18, 19]. Ablation also produces histological and cytological alterations—hyalinization, necrosis, hemorrhage, calcifications, and treatment‐related atypia—that can mimic or obscure diagnostic features, particularly in follicular‐patterned lesions, complicating the assessment of capsular and vascular invasion and potentially affecting Bethesda categorization [15, 16, 20].

Against this background, there is a need to better define how prior thermal ablation affects both the surgical and pathological aspects of thyroidectomy [13, 14]. This study aims to characterize perioperative outcomes of thyroidectomy after RFA, focusing on operative difficulty, adhesion patterns, and complication rates, and to describe the extent of ablation‐related histopathological changes. By correlating clinical, ultrasonographic, and pathological parameters with operative complexity and postoperative morbidity, the study seeks to identify risk factors for challenging surgery and diagnostic uncertainty, thereby informing patient selection, counseling, and decisions between repeat ablation and upfront surgery.

## 2 Materials and Methods

### 2.1 Study Design and Institutional Setting

This retrospective cohort study included all consecutive patients who underwent thyroid surgery between January 2021 and December 2025, identified through a prospectively maintained institutional registry at the Department of Surgery, University of Milan, Istituto Auxologico Italiano (Milan, Italy). The institution is a high‐volume endocrine surgery center with integrated multidisciplinary thyroid care and recognized national and international referral activity. All included patients had undergone at least one previous minimally invasive thyroid ablative procedure before surgery. This study was reported in accordance with the STROBE guidelines for observational cohort studies.

### 2.2 Ethics Approval and Informed Consent

This study was conducted in accordance with the Declaration of Helsinki and applicable institutional policies governing retrospective observational research. Given the retrospective design and exclusive use of data from routine clinical care, the requirement for study‐specific informed consent was waived. At the institution, all patients undergoing thyroid surgery routinely provide broad consent authorizing the use of anonymized clinical and pathological data for scientific and educational purposes. The study protocol (approval No. 2.792.414) received prior approval from the Institutional Ethics Committee.

### 2.3 Eligibility Criteria and Case Definition

Eligible ablative modalities included percutaneous ethanol injection and radiofrequency ablation for thyroid nodular disease. Inclusion criteria were as follows: at least one prior minimally invasive ablative procedure; complete clinical, radiological, surgical, and pathological data; an indication for surgery consistent with ATA recommendations (compressive or cosmetic symptoms, nodular regrowth, or oncological suspicion, including suspicious cytology, suspected medullary carcinoma, or Graves' disease); availability of an intact specimen for full histopathological reassessment; and surgery and pathology performed at the study institution [21]. Preoperative laryngoscopic examination was performed on all patients. Exclusion criteria were prior thyroid surgery; malignant diagnosis before ablation; incomplete documentation; previous head–neck radiotherapy; prior radioiodine therapy; or documented nodule rupture during ablation or macroscopic capsular disruption at thyroidectomy. All patients who underwent hemithyroidectomy received ablation on the same side as the resected lobe; patients who had ablation only on the contralateral lobe were excluded.

### 2.4 Demographic and Preoperative Clinical Characteristics

Demographic variables included age at the time of thyroid surgery and sex. Preoperative laboratory evaluation included serum thyroid‐stimulating hormone (TSH), free thyroxine (fT4), free triiodothyronine (fT3), antithyroid peroxidase antibodies (anti‐TPO), antithyroglobulin antibodies (anti‐Tg), thyroglobulin (Tg), and serum calcitonin. Preoperative clinical characteristics included total thyroid volume, the presence of preoperative suspicion of malignancy (yes/no), and the primary indication for surgery. Surgical indications were classified as compressive symptoms, cosmetic or esthetic concern, nodular regrowth following thermal ablation, cytological suspicion of malignancy on fine‐needle aspiration, suspected medullary thyroid carcinoma based on elevated calcitonin levels, or hyperthyroidism due to Graves' disease [21].

### 2.5 Ablation‐Related Variables

Ablation‐related variables were systematically recorded and included the ablation modality (radiofrequency or ethanol ablation), the number of thyroid nodules treated per patient, and the largest diameter of the index (surgically resected) nodule at the time of ablation. For each patient, the number of ablation sessions (1 or 2 or more), the percentage volume reduction of the treated nodule after ablation (when available), and the interval between the last ablation session and subsequent surgery (expressed in months) were also collected. In accordance with the institutional protocol, all patients underwent at least two separate fine‐needle aspiration cytology (FNAC) assessments prior to the ablative procedure to confirm benign or low‐risk cytological characteristics [5, 6, 7].

### 2.6 Intraoperative and Postoperative Surgical Outcomes

Surgical variables included the type of thyroidectomy (hemithyroidectomy, total, or staged completion), operative time (separately for hemi and total procedures), intraoperative blood loss, and nodule size at surgery. Neuromonitoring parameters included recurrent laryngeal nerve (RLN) loss of signal (LOS) and conduction impairment, defined respectively as absent or < 100 μV evoked amplitude despite standard stimulation, and as ≥ 50% amplitude reduction with ≥ 10% latency increase without complete LOS [22]. Postoperative outcomes were RLN palsy, cervical hematoma requiring reoperation, and hypoparathyroidism, defined by serum PTH < 13 pg/mL. Serum parathyroid hormone (PTH) levels were measured within 24 h after surgery (typically 6–12 h postoperatively) using a second‐generation immunoassay with a normal reference range of 15–65 pg/mL; in accordance with recent international consensus, transient hypoparathyroidism was defined as PTH < 13 pg/mL with subsequent recovery within 6 months, whereas permanent hypoparathyroidism was defined as persistently subnormal PTH levels beyond 6 months after thyroidectomy [23]. RLN palsy was classified as permanent when vocal cord dysfunction persisted for more than 6 months after surgery. Patients were classified by the presence or absence of ≥ 1 postoperative complication. RLN injury and hypoparathyroidism were considered transient if resolving within 6 months and permanent thereafter [24, 25]. Incidental parathyroidectomy on final pathology was also recorded.

All procedures were performed with intermittent intraoperative neuromonitoring using the C2 NerveMonitor (inomed Medizintechnik GmbH, Emmendingen, Germany), whereas continuous intraoperative neuromonitoring (C‐IONM) was not employed in this cohort.

### 2.7 Histopathological Assessment of Post‐Ablative Thyroid Specimens

In accordance with standard surgical and oncological practice, thyroid nodules were not clamped, grasped, or incised intraoperatively and the entire specimen was submitted intact for pathological assessment. All specimens were reevaluated by two endocrine pathologists (GG and AS), with specific attention to the previously ablated nodule. Histopathological assessment included documentation of thyroiditis, the presence of multiple nodules, and detailed characterization of the dominant (index) nodule according to established criteria [21, 25].

Quantitative morphometric assessment focused on three primary components within the treated nodule—sclerosis, coagulative necrosis, and residual viability—defined respectively as the proportions of mature fibrotic tissue, acellular eosinophilic necrosis, and remaining viable thyroid parenchyma [26]. For each case, representative full‐face sections of the index nodule were examined on hematoxylin–eosin–stained slides. The relative proportion of each component was estimated visually by experienced endocrine pathologists using low‐ and intermediate‐power fields, integrating the overall assessment across the entire nodule section. Percentages were assigned in approximate 5%–10% increments and expressed as the proportion of the total nodule area, with the sum of the three components accounting for 100%.

Sclerosis was defined as mature collagenized fibrosis with distortion or loss of normal follicular architecture; coagulative necrosis as acellular eosinophilic areas with preservation of tissue outlines; and residual viability as preserved thyroid parenchyma with intact follicular structures. In cases of relevant discrepancy between observers, a joint review was performed and a consensus estimate was recorded.

A composite maturation index integrating these elements was calculated (Supporting Information S1). Additional features recorded included capsule presence and thickness, the regularity of the nodule–parenchyma interface, minimum nodule‐to‐capsule distance (< 1 mm when applicable), capsular alterations, and perithyroidal soft‐tissue changes (hemorrhage, chronic inflammation, and fibrosis) [26]. The presence and location of concomitant carcinoma were also noted. Immunohistochemistry (HBME‐1, Galectin‐3, CK19, BRAF V600E, as appropriate) was performed according to routine diagnostic protocols to support tumor classification and interpretation of treatment‐related cytological changes [21].

### 2.8 Maturation Index

Two experienced pathologists (GG and AS), blinded to all clinical, surgical, and outcome data, independently evaluated the histological specimens.

For each case, the relative proportion of sclerosis/fibrosis, coagulative necrosis, and residual viable tissue was estimated on routine hematoxylin–eosin–stained sections and expressed as a percentage of the total tissue area, with the sum of the three components accounting for 100% of the analyzed section.

In cases of relevant discrepancy between observers, a joint review was performed and a consensus estimate was recorded. Interobserver agreement was explored descriptively in this initial series and will be formally assessed in a subsequent validation cohort.

Based on these quantitative estimates, an exploratory histological maturation index was calculated as follows:

(Sclerosis+Vitality)/(Necrosis+1)

where a constant was added to the denominator to avoid division by zero.

This index was conceived as a pragmatic composite descriptor of postablation tissue remodeling and was not intended as a validated scoring system.

### 2.9 Comparative Analysis With a Contemporaneous Nonablated Thyroidectomy Cohort

A contemporaneous institutional cohort of patients undergoing thyroidectomy without prior ablation was used solely to provide descriptive clinical context. This comparison was not intended for causal inference as no matching or risk adjustment was performed and the groups differed in baseline characteristics and surgical indications.

Accordingly, between‐cohort data are presented descriptively. The reference cohort included patients with no history of thermal or chemical ablation and was analyzed using identical definitions of surgical procedures and postoperative complications. Complications were recorded at discharge and follow‐up and classified as recurrent laryngeal nerve injury, hypoparathyroidism, and cervical hematoma requiring reoperation.

### 2.10 Statistical Analysis

Given the limited sample size and non‐normal distribution of several variables, nonparametric statistical methods were used. No a priori sample size calculation was performed; the cohort reflects all eligible cases during the study period. Continuous variables (sclerosis, necrosis, residual viability, maturation index, and ablation‐to‐surgery interval) were compared between patients with and without complications using the Mann–Whitney *U* test. Categorical variables, including concomitant carcinoma and discrete histological features, were analyzed with Fisher's exact test. Correlations between histopathological parameters and time from ablation were assessed using Spearman's *ρ*. All analyses were exploratory, with *p*‐values interpreted descriptively and without correction for multiple testing. Potential biases related to retrospective referral patterns and incomplete preablation data were mitigated through predefined eligibility criteria and complete‐case analysis.

Correlations between morphometric parameters (sclerosis, necrosis, residual viability, and maturation index) and ablation‐related variables, including ablation‐to‐surgery interval and nodule size at ablation, were explored using Spearman's rank correlation coefficient.

Corresponding scatter plots with trend visualization are provided in the Supporting Information. Given the exploratory nature of these analyses and the limited sample size, *p*‐values were interpreted descriptively.

## 3 Results

### 3.1 Demographic and Preoperative Characteristics

**FIGURE 1:**
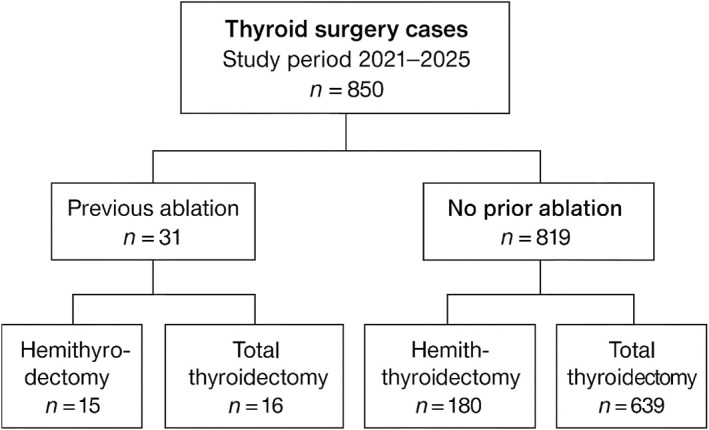
Flowchart of the study cohort.

The patient selection process is shown in Figure 1. Thirty‐one patients who underwent thermal ablation followed by thyroid surgery were included, all operated under biochemical euthyroidism (Table 1). Case distribution over time is presented in Supporting Information S2: Figure S1. The mean age was 52.5 ± 11.3 years (median 53 and IQR 47–58), and most patients were female (24/31 and 77.4%). Surgical indications included multinodular goiter (58.1%), uninodular goiter (22.6%), suspicious cytology (12.9%), and, less frequently, suspected medullary carcinoma or Graves' disease (3.2% each) (Supporting Information S2: Figure S2). Preoperative laryngoscopy showed normal vocal cord mobility in all patients, with no cases of preexisting vocal cord dysfunction identified. Preoperative suspicion of malignancy was present in 16.1% of cases. The mean maximum diameter of the previously ablated nodule at surgery was 42.8 mm (median 42.5 mm and IQR 34.6–50.0), and mean thyroid volume was 58.0 mL (median 53.0 mL and IQR 42.6–68.3). The interval between ablation and surgery averaged 43.9 months (median 35 and IQR 17.5–63.5), reflecting substantial heterogeneity (Supporting Information S2: Figures S3 and S4).

**TABLE 1:** Demographic and preoperative clinical characteristics (*n* = 31).

Variable	Median (IQR)	*n* (%)
Age (years)	53 (47–58)	—
F	—	24 (77.4)
M	—	7 (22.6)
Suspicion of malignancy (preoperative)	—	5 (16.1)
Nodule size	42.5 mm (34.6–50.0)	—
Thyroid volume	53 mL (42.6–68.3)	—
Ablation–surgery interval	35 months (17.5–63.5)	—
**SURGICAL INDICATIONS**		
Multinodular goiter (MNG)	—	18 (58.1)
Uninodular goiter (UNG)	—	7 (22.6)
Suspicious cytology	—	4 (12.9)
Suspected medullary carcinoma	—	1 (3.2)
Graves' disease	—	1 (3.2)

### 3.2 Ablation Characteristics and Treatment Response

At the time of the first ablative procedure, the median maximum diameter of the treated nodule was 45 mm (IQR 41–50) (Supporting Information S2: Table S1). Most patients (26/31 and 83.9%) underwent a single ablation session, whereas 5 patients (16.1%) required a second session. Radiofrequency thermal ablation was the most frequently used modality (21 patients, 67.7%), followed by percutaneous ethanol injection (9 patients, 29.0%); in 1 case (3.2%), both techniques were used in combination. No patients in the final study cohort had undergone microwave ablation; therefore, outcomes related to this modality were not evaluated. Treatment response was measured as the percentage change in nodule size relative to baseline (negative values indicating reduction and positive values indicating increase) and was available for 18 patients (58.1%). Overall, the relative size change was modest, with a median relative change of −0.15 (IQR −0.43 to +0.16) and a mean of −0.16 ± 0.46, indicating a general tendency towards size reduction but with substantial interpatient variability, including cases of interval nodule enlargement after ablation (Supporting Information S2: Figure S5). In our analysis, no patients underwent ablation exclusively on the contralateral lobe and no patient underwent bilateral ablation of distinct nodules before surgery; all ablation procedures were limited to a single dominant nodule or a single multinodular lobe.

### 3.3 Perioperative Outcomes, RLN Neuromonitoring Profiles, and Complications Following Previous Thyroid Ablation

**FIGURE 2:**
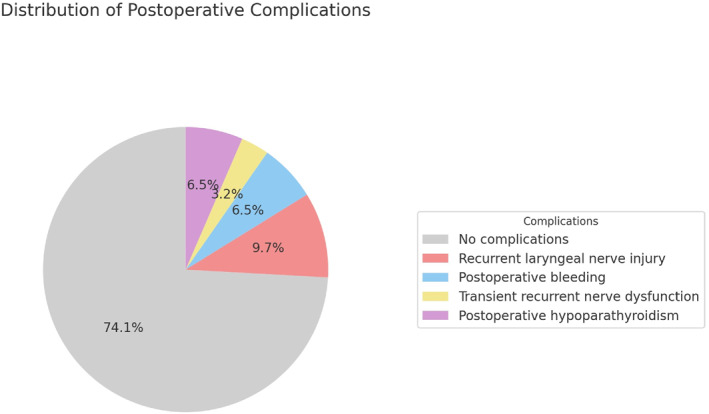
Distribution of postoperative complications in the study cohort. The pie chart shows the proportion of patients with no complications compared to those with recurrent laryngeal nerve injury, postoperative bleeding, transient recurrent nerve dysfunction, and postoperative hypoparathyroidism.

**FIGURE 3:**
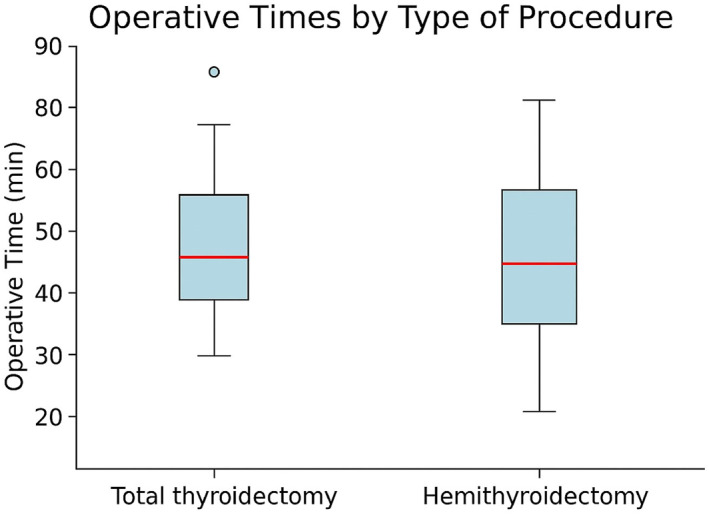
Operative times by type of procedure. Boxplots compare the distribution of operative duration (minutes) between total thyroidectomy and hemithyroidectomy, showing the median values, interquartile ranges, and outliers for each surgical group.

Of the 31 patients, 16 (51.6%) underwent total thyroidectomy and 15 (48.4%) underwent hemithyroidectomy. The median operative time was 50 min (IQR 45–61.25) for total thyroidectomy and 40 min (IQR 35–60) for hemithyroidectomy. Postoperative outcomes were favorable in most cases, with 23 patients (74.2%) experiencing no complications (Figures 2 and 3). Intraoperative loss of signal (LOS) of the recurrent laryngeal nerve (RLN) was documented in 5 patients (16.1%), corresponding to 5 of 47 nerves at risk (NAR) (10.6%). In one of these patients (3.2%), LOS led to a planned two‐stage thyroidectomy. Intraoperative RLN conduction impairment, as defined in the Methods section, was observed in 1 patient (3.2%). Overall, 6 of 31 patients (19.4%)—6 of 47 NAR (12.8%)—experienced some degree of intraoperative RLN functional deterioration (LOS or conduction impairment). Postoperative laryngoscopy documented RLN palsy in 3 patients (9.7%). Additional complications included postoperative cervical hematoma requiring reoperation in 2 patients (6.5%) and postoperative hypoparathyroidism in 2 patients (6.5%). No permanent complications were observed according to the predefined criteria (Table 2).

**TABLE 2:** Surgical procedures and postoperative complications in the postablation cohort.

Variable	*n*	%	Median	IQR
Total thyroidectomy	16	51.6	—	—
Operative time	—	—	50 min	45–61.25
Hemithyroidectomy	15	48.4	—	—
Operative time	—	—	40 min	35–60
Loss of RLN signal	5	16.1	—	—
Two‐stage thyroidectomy	1	3.2	—	—
Complications				
Recurrent laryngeal nerve injury	3	9.7	—	—
Postoperative bleeding requiring reoperation	2	6.5	—	—
Transient recurrent nerve dysfunction	1	3.2	—	—
Postoperative hypoparathyroidism (PTH < 13 pg/mL)	2	6.5	—	—
Permanent complications (> 6 months)	0	—	—	—

### 3.4 Histopathological Findings

**FIGURE 4:**
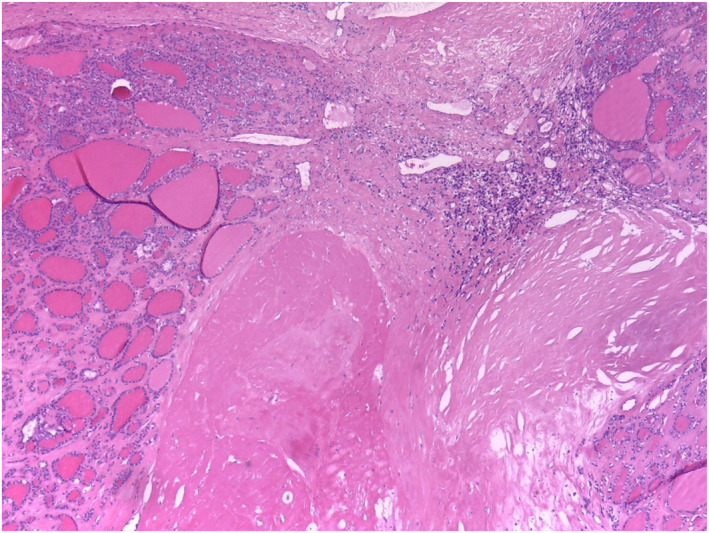
Histopathological section of a postablation thyroid nodule showing a broad central area of coagulative necrosis, surrounded by residual viable follicles and a peripheral rim of fibroinflammatory reaction. The interface between necrotic tissue and preserved thyroid parenchyma highlights the zonal pattern of thermal injury and early reparative changes.

**FIGURE 5:**
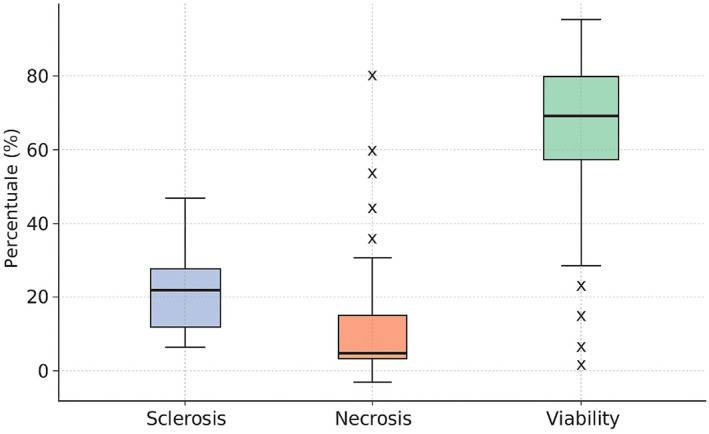
Boxplots showing the percentage distribution of sclerosis, coagulative necrosis, and residual cellular viability within ablated thyroid nodules. The plots display median values, interquartile ranges, whiskers, and outliers for each histological component, highlighting the considerable variability in tissue remodeling after ablation.

Within previously ablated nodules, three main histological components were quantified—sclerosis, coagulative necrosis, and residual viable tissue—representing fibrotic, necrotic, and preserved parenchymal elements, respectively (Figure 4). Mean values were 19.2% sclerosis, 14.6% necrosis, and 66.1% residual viability (Supporting Information S2: Table S2a,b), indicating a predominance of viable tissue over fibrotic or necrotic components. Additional features included a capsule in all assessable cases (thin in 77.4% and thick in 16.1%), a regular nodule–parenchyma interface in 61.3%, and nodule‐to‐capsule distance < 1 mm in 77%. Capsular alterations were rare (6.5%). Perithyroidal soft‐tissue changes were present in 48.4% of specimens, most commonly mild chronic inflammation with hemorrhagic extravasation (46.7%) or isolated hemorrhage (40%), with less frequent fibrosis‐associated patterns (Figure 5). These findings are consistent with reactive postablative changes rather than primary disease‐specific lesions.

Correlation analyses between morphometric parameters and ablation‐related variables are shown in Supporting Information S2: Figures S6–S9. No significant association was observed between sclerosis and either ablation‐to‐surgery interval (*ρ* = −0.20 and *p* = 0.29) or nodule size (*ρ* = −0.12 and *p* = 0.59). The maturation index showed a moderate correlation with ablation‐to‐surgery interval (*ρ* = 0.47 and *p* = 0.010), whereas no significant correlation with nodule size was detected (*ρ* = 0.28 and *p* = 0.19).

Incidental malignancies included two papillary microcarcinomas (6.4%) and one medullary carcinoma (3.2%). None of these carcinomas arose within the previously ablated index nodule; all three were considered topographically and morphologically unrelated to the treated lesion. For each of these cases, Supporting Information S2: Table S4 summarizes histologic subtype, whether a malignant nodule was sampled at preablation FNA, the percentage volume change of the ablated nodule when available, and the primary indication for surgery (suspicious cytology, elevated calcitonin, or multinodular goiter).

### 3.5 Risk Factors for Surgical Complications: Exploratory Analysis

To identify histopathological correlates of surgical morbidity, quantitative tissue variables were examined in an exploratory analytical framework (*n* = 31). Owing to sample size constraints, analyses focused on bivariate associations, derived indices, and ROC‐based cut‐offs rather than fully powered multivariable models. Among all morphometric parameters, sclerosis was the only significant predictor of complications: patients with postoperative events showed higher mean sclerosis than uncomplicated cases (30.0% vs. 16.9% and *p* = 0.008), whereas nodule size, ablation‐to‐surgery interval, and concomitant carcinoma were not associated with risk. The maturation index ((sclerosis + viability)/(necrosis + 1)) correlated with time from ablation (*ρ* = 0.47 and *p* = 0.010), indicating progressive fibrotic remodeling, but did not differentiate between patients with and without complications (*p* = 0.32) (Supporting Information S2: Figure S4). A simplified fibrosis metric (sclerosis—necrosis) identified an ROC‐derived threshold of ∼20% points distinguishing patients at higher complication risk. Although exploratory, these findings support a model in which advanced stromal reorganization—predominantly mature fibrosis rather than elapsed time—represents the principal histological driver of operative difficulty and postoperative morbidity.

Relative size change response data were available for 18 of the 31 patients (58.1%) and were included in an exploratory subanalysis. We assessed the association between percentage nodule volume change after radiofrequency ablation and a composite endpoint of postoperative complications (any recurrent laryngeal nerve palsy, hypoparathyroidism, or cervical hematoma). Percentage volume reduction did not differ significantly between patients with and without complications (Mann–Whitney *U* test and *p* = 0.756).

## 4 Discussion

This study provides the first quantitative clinicopathological analysis directly linking postablative histological remodeling with operative difficulty and perioperative morbidity. By measuring the proportions of sclerosis, coagulative necrosis, and residual viability, we show that fibrosis—rather than nodule size or time from ablation—is the principal tissue‐level determinant of surgical risk. Higher sclerosis was strongly associated with complications, supporting the hypothesis that mature scar tissue creates rigid, adherent planes that impair safe dissection around the RLN and major vessels. Both the fibrosis‐weighted metric (sclerosis–necrosis) and the maturation index suggest a potential “fibrotic threshold” beyond which thyroidectomy becomes technically challenging, although these indices remain exploratory and require external validation. Despite the modest sample size, this represents one of the largest series with paired quantitative pathology and the magnitude of the observed effects—particularly for sclerosis—was sufficient to emerge even with limited statistical power.

From a surgical perspective, the findings suggest that thyroidectomy after radiofrequency or chemical ablation should be considered a specialized procedure performed in high‐volume endocrine units with expertise in complex and reoperative neck surgery [3]. Ablation‐related scarring often distorts normal anatomy, tethers the thyroid capsule to surrounding soft tissues, and may extend along the tracheoesophageal groove, thereby increasing the risk of traction, thermal, or clamp injury to the recurrent laryngeal nerve and complicating hemostasis. In these circumstances, systematic intraoperative neuromonitoring and advanced visualization technologies are key risk‐mitigation tools, enabling early recognition of nerve stress, loss of signal, or conduction impairment and facilitating staged strategies when electrophysiological deterioration occurs. Centralization of postablative thyroidectomy in centers where such technology and experience are routinely available is justified by the observed pattern of complications [2, 3].

To provide clinical context, we compared perioperative outcomes in the ablation cohort with those observed in contemporaneous thyroidectomies performed at our institution. This comparison was not designed for formal causal inference as no matching or adjustment was performed and the two populations differed in baseline characteristics and indications for surgery.

Within these limitations, ablated patients showed numerically higher rates of overall complications (22.6% of ablated patients vs. 12.9% of controls) and recurrent laryngeal nerve palsy (9.7% vs. 4.6%; 6.4% vs. 2.6% per nerve at risk) as well as a higher incidence of postoperative hematoma (6.5% of ablated patients vs. 1.8% of controls), whereas hypoparathyroidism rates were comparable (6.5% vs. 6.6%). Although none of these differences reached statistical significance, the observed trends are compatible with the altered tissue planes and fibrotic remodeling documented histologically.

These findings suggest that thyroidectomy after ablation is technically feasible in experienced centers but may be associated with modestly increased procedural complexity. They should be interpreted as hypothesis‐generating and underscore the importance of meticulous dissection, routine neuromonitoring, and careful postoperative surveillance.

This work complements and extends the report by Kuo et al., who, in a smaller cohort, documented longer operative times, higher difficulty and adhesion scores, and increased rates of incidental parathyroidectomy in thyroidectomy after thermal ablation, along with a qualitative description of ablation‐related histological changes that may obscure capsule integrity and complicate the diagnosis of follicular‐patterned neoplasms [19]. Although that study focused primarily on macroscopic difficulty metrics and descriptive pathology, the present analysis adds a layer of quantitative morphometrics and demonstrates that mature fibrosis, rather than the mere fact of prior ablation or the ablation‐to‐surgery interval, is the dominant histopathological correlate of RLN injury and postoperative bleeding. In this respect, macroscopic adhesion and difficulty scores and the microscopic, index‐based approach proposed here are methodologically complementary: the former identify high‐risk procedures intraoperatively, whereas the latter provide a biologically grounded framework for understanding why certain postablative fields are particularly hazardous [17].

The study also highlights the pivotal role of expert endocrine pathology in managing these patients. Ablation‐related hyalinization, necrosis, hemorrhage, and treatment‐related atypia can both mimic and mask malignancy, and clinically relevant neoplasms—including incidental microcarcinomas and medullary carcinoma—were identified in a significant proportion of specimens. Detailed, standardized sampling and reporting of the entire resected lobe or gland are therefore essential, both for accurate tumor detection and classification and for medico‐legal robustness in the context of postoperative complications. Quantitative reporting of sclerosis, necrosis, and residual viability, documentation of capsular and perithyroidal alterations, and judicious use of immunohistochemistry are particularly important to distinguish treatment effects from true invasive disease and to maintain transparent clinicopathological correlation.

The maturation index introduced in this study represents a novel disease‐specific composite descriptor designed to summarize the balance between chronic reparative changes (sclerosis/fibrosis), destructive injury (necrosis), and preservation of residual viable tissue within a single quantitative metric. Supporting Information S1 correlation analyses indicated that the maturation index increased with time after ablation, reflecting progressive tissue remodeling. In contrast, sclerosis, which was the only parameter associated with postoperative complications, did not correlate with either time interval or nodule size.

These findings suggest that clinically relevant fibrotic changes are not simply time‐dependent or volume‐driven, but likely reflect heterogeneous patient‐specific remodeling processes. Given the small sample size and exploratory design, these results should be interpreted cautiously and warrant confirmation in larger prospective cohorts.

An important unanswered question concerns which patients are more likely to develop fibrosis‐dominant rather than necrosis‐dominant remodeling after ablation. From a clinical perspective, the ability to anticipate these patterns preoperatively could be valuable for surgical planning and risk stratification. In the present study, exploratory descriptive analyses did not support reliable identification of clinical or procedural predictors of preferential fibrotic or necrotic response. This is most likely attributable to the limited sample size and the marked interindividual heterogeneity observed. Accordingly, the present data do not allow development of robust classification or prediction models.

Future prospective studies with larger cohorts standardized imaging follow‐up, and integrated biological profiling will be required to clarify determinants of postablation remodeling patterns and to evaluate their potential role in guiding clinical decision‐making. Although similar multiparametric histopathological approaches integrating fibrosis, necrosis, and tissue viability have been adopted in other clinical contexts, including chronic hepatitis grading–staging systems, tumor regression grading, and ischemic skeletal muscle injury scores, the present index constitutes a context‐adapted construct specifically developed for postablation thyroid tissue [27, 28, 29].

Accordingly, it should be regarded as exploratory and hypothesis‐generating. In the present study, it was primarily used to describe patterns of tissue remodeling and to support mechanistic interpretation of surgical outcomes. Formal validation in larger independent cohorts will be required to assess inter and intraobserver reproducibility, establish correlations with established clinicopathologic endpoints and determine whether specific index thresholds are associated with clinically meaningful differences in risk or prognosis.

Three malignancies were detected within this cohort (two papillary microcarcinomas and one medullary carcinoma). As detailed in Supporting Information S2: Table S4, they did not originate within the previously ablated nodules and were instead considered unrelated synchronous lesions. This observation suggests that, in our series, radiofrequency or ethanol ablation was not implicated in the malignant transformation of the treated index nodules, although the small number of events precludes firm conclusions. Nevertheless, these findings reinforce the need for rigorous preablation cytologic assessment and continued surveillance of the entire thyroid gland, including nonablated nodules, particularly in patients with suspicious cytology or elevated calcitonin.

This study has several limitations. The cohort size is modest, reflecting the rarity of post‐ablative thyroidectomy in a single high‐volume center, and limits statistical power; accordingly, fibrosis‐based thresholds (e.g., sclerosis–necrosis differential and maturation index) should be considered exploratory. The retrospective design may introduce selection bias as patients referred for surgery after ablation likely represent a clinically enriched subset, limiting generalizability. Morphometric assessment was based on semiquantitative visual estimation by a single pathology team, which mirrors real‐world practice but may involve interobserver variability compared with digital image analysis. Comparison with the contemporaneous nonablated cohort is contextual only and cannot adjust for unmeasured differences in case mix, and multivariable modeling was not feasible given the low event count. Intraoperative difficulty was inferred indirectly from operative time, complications, and neuromonitoring findings rather than a validated adhesion score. Finally, all procedures were performed in a tertiary endocrine center with routine neuromonitoring and specialized pathology, and external validation in multicenter settings is required. In terms of neurominitoring, C‐IONM was not used; therefore, outcomes could not be compared with those of a contemporaneous group managed with C‐IONM. Given the increased technical complexity of thyroidectomy after ablation, the use of C‐IONM would be particularly appealing, as it has been shown to be superior to intermittent IONM in reducing the risk of vocal cord palsy. However, in our setting, C‐IONM was not implemented for these patients because of economic constraints [30].

## Author Contributions

**Daqi Zhang:** conceptualization, methodology, data curation, formal analysis, writing – original draft. **Alvino Boero:** investigation, data curation, writing – review and editing. **Giacomo Gazzano:** investigation, resources, data curation. **Andrea Satta:** investigation, resources, data curation. **Laura Fugazzola:** supervision, validation, writing – review and editing. **Carla Colombo:** supervision, validation, writing – review and editing. **Francesco Brucchi:** conceptualization, methodology, project administration, writing – review and editing. **Gianlorenzo Dionigi:** conceptualization, supervision, project administration, writing – review and editing.

## Funding

The authors have nothing to report.

## Conflicts of Interest

The authors declare no conflicts of interest.

## Supporting information


Supporting Information S1

Supporting Information S2

## Data Availability

The data that support the findings of this study are available from the corresponding author upon request. The data are not publicly available due to privacy or ethical restrictions.
