# Supplementary material for: Fibrosis‐Driven Surgical Risk After Thyroid Nodule Ablation: Quantitative Clinicopathological Determinants of Complications in Post‐Ablative Thyroidectomy—A Retrospective Cohort Study
Source: World J Surg. 2026 Mar 8;50(4):914–23. doi: 10.1002/wjs.70300 (PMC13070446; doi:10.1002/wjs.70300)
Supplement: Supplementary file 1 — Supporting Information S1 [file WJS-50-914-s002.pdf]

Supplementary material 1. Maturation index

A maturation index was developed to provide an integrated quantitative measure of post-ablative tissue remodelling within each resected thyroid nodule. This index was designed to capture, in a single parameter, the relative contributions of reparative fibrosis, preserved parenchyma, and necrotic damage, thereby reflecting the histological "stage" of healing after thermal injury. For each case, three morphometric components were quantified on routine haematoxylin–eosin–stained sections: (1) the percentage of the nodular area occupied by sclerosis (mature fibrocollagenous tissue), (2) the percentage composed of coagulative necrosis, and (3) the percentage represented by residual viable thyroid follicles (residual cellular viability). All measurements were expressed as a proportion of total nodule area, assessed by semi-quantitative visual estimation or image-assisted morphometry according to local practice.

The maturation index was then calculated as

$$\text{Maturation index} = \frac{\text{Sclerosis} + \text{Residual viability}}{\text{Coagulative necrosis} + 1}$$

In this formulation, the numerator combines the two components that indicate structurally organised tissue—mature scar and surviving parenchyma—while the denominator represents the burden of non-viable necrotic tissue. Higher values of the index therefore correspond to a more advanced stage of maturation, characterised by predominance of fibrosis and/or viable follicles over coagulative necrosis, whereas lower values reflect a histological pattern dominated by necrotic debris.

The constant "+1" was added to the denominator for two reasons. First, it prevents mathematical indeterminacy in samples with absent or negligible coagulative necrosis, where the raw percentage would otherwise be zero. Second, it minimises instability of the index in nodules with very small necrotic fractions, in which minor measurement variability could disproportionately inflate the calculated value. By construction, the maturation index thus provides a continuous, dimensionless variable that is robust across the full spectrum of observed necrosis, from 0% to high-burden lesions. This parameter was subsequently used in exploratory analyses as a surrogate marker of the overall histological state of post-ablative remodelling and its potential relationship with operative difficulty and postoperative outcomes.
