# Supplementary material for: Fibrosis‐Driven Surgical Risk After Thyroid Nodule Ablation: Quantitative Clinicopathological Determinants of Complications in Post‐Ablative Thyroidectomy—A Retrospective Cohort Study
Source: World J Surg. 2026 Mar 8;50(4):914–23. doi: 10.1002/wjs.70300 (PMC13070446; doi:10.1002/wjs.70300)
Supplement: Supplementary file 2 — Supporting Information S2 [file WJS-50-914-s001.docx]

Figure 1s: The plot shows a progressive increase in the number of patients treated over the study period, with a steeper rise in cumulative cases in recent years.


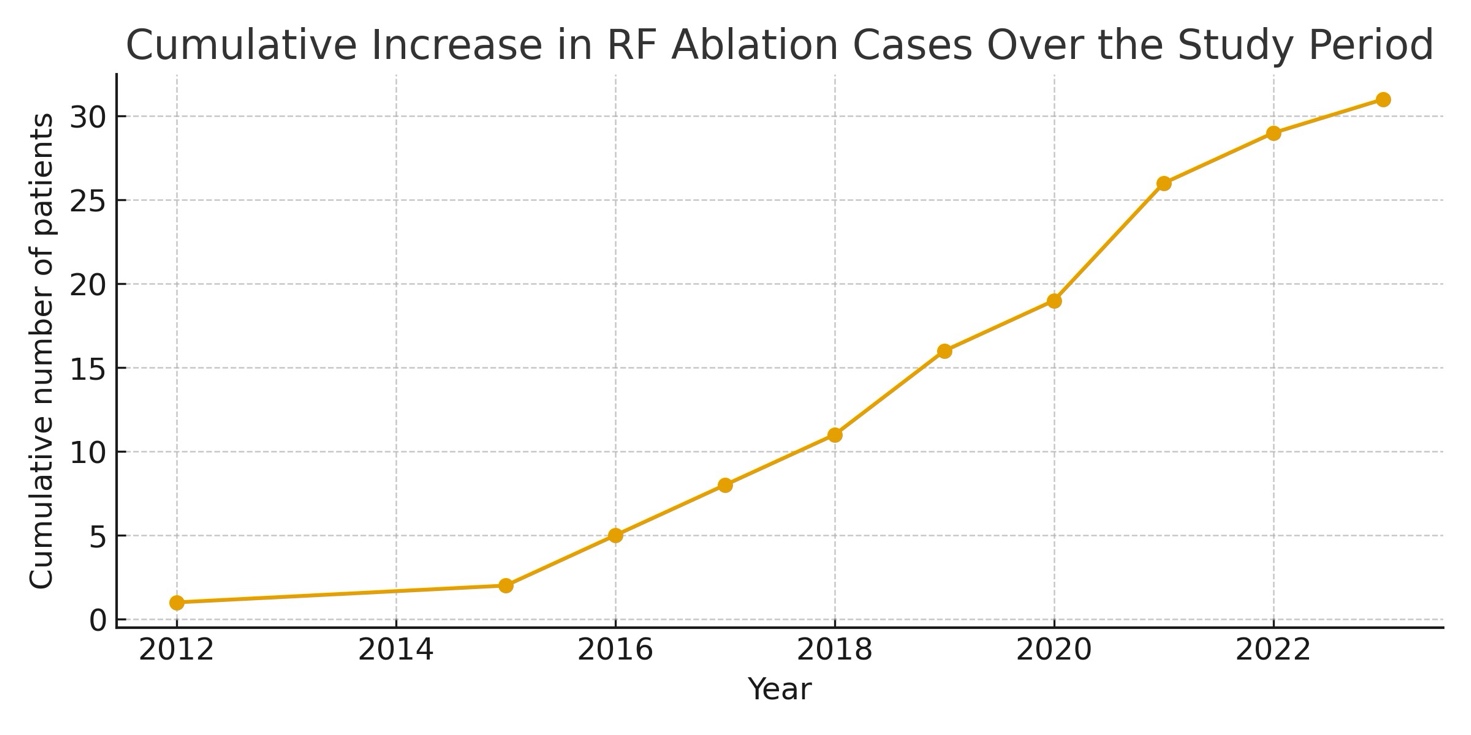


Figure 2s: Distribution of surgical indications and preoperative suspicion of malignancy in patients previously treated with radiofrequency ablation. Multinodular goitre (MNG) was the most common indication, followed by uninodular goitre (UNG), suspicious cytology, suspected medullary carcinoma, and Basedow disease. The red bar highlights the subgroup with a documented preoperative suspicion of malignancy.


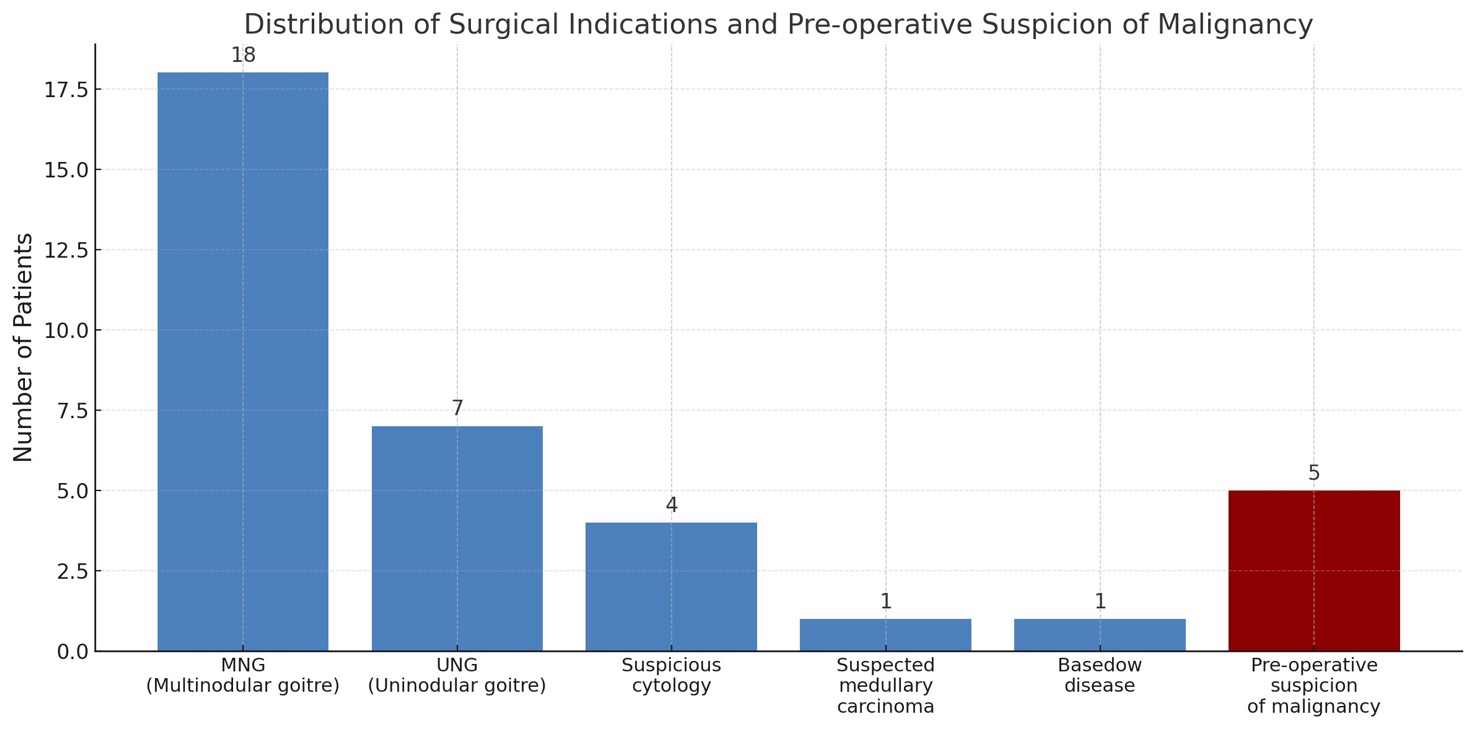


Figure 3s: Boxplots illustrating nodule size at surgery (left) and the interval between ablation and surgery in months (right). Both distributions show substantial variability, with several outliers reflecting the heterogeneity of the treated population.


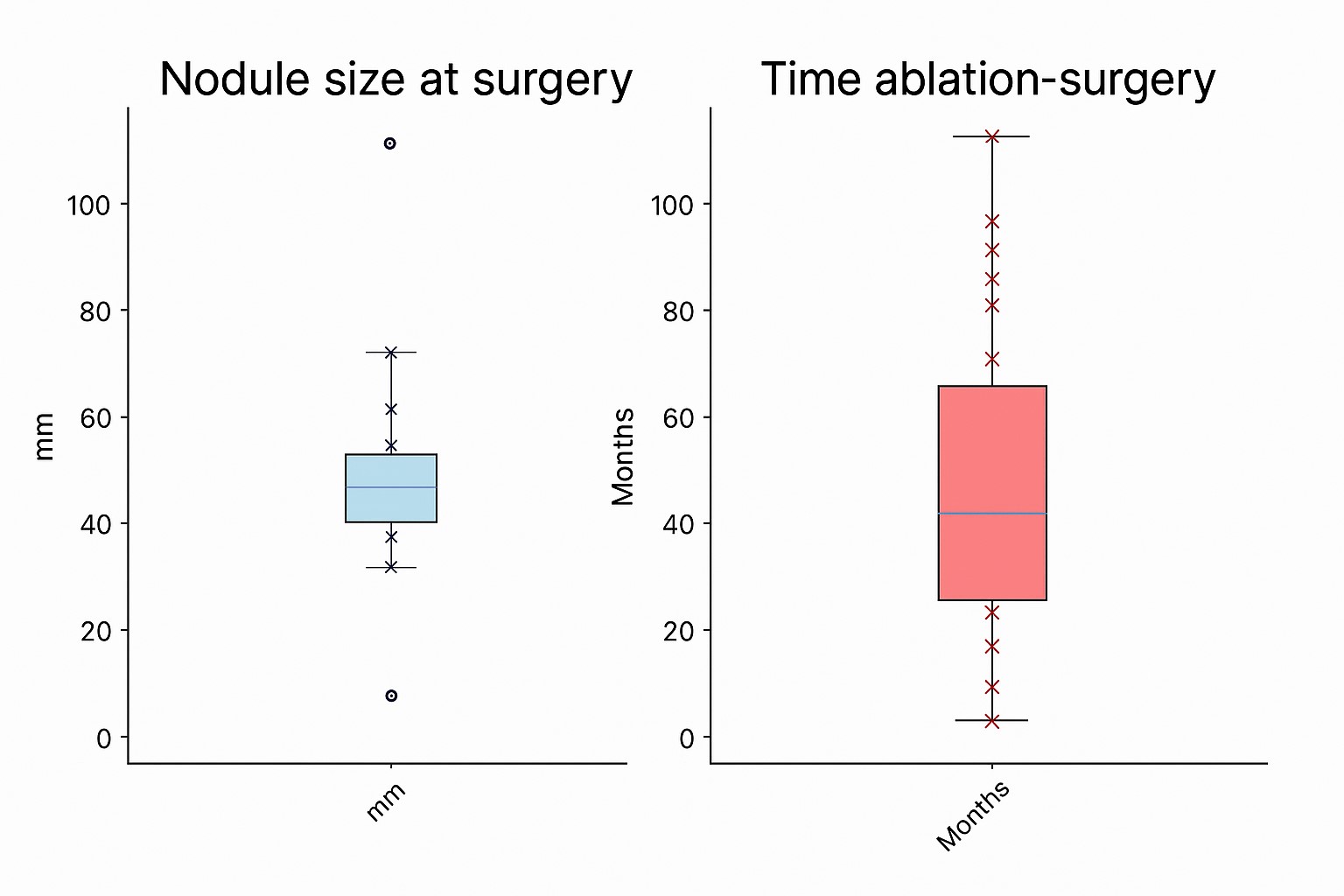


Figure 4s: Histogram showing the distribution of patients by the time interval between radiofrequency (RF) ablation and surgery, with most cases clustered within the first 30 months and several long-interval outliers indicating substantial variability in follow-up timing.


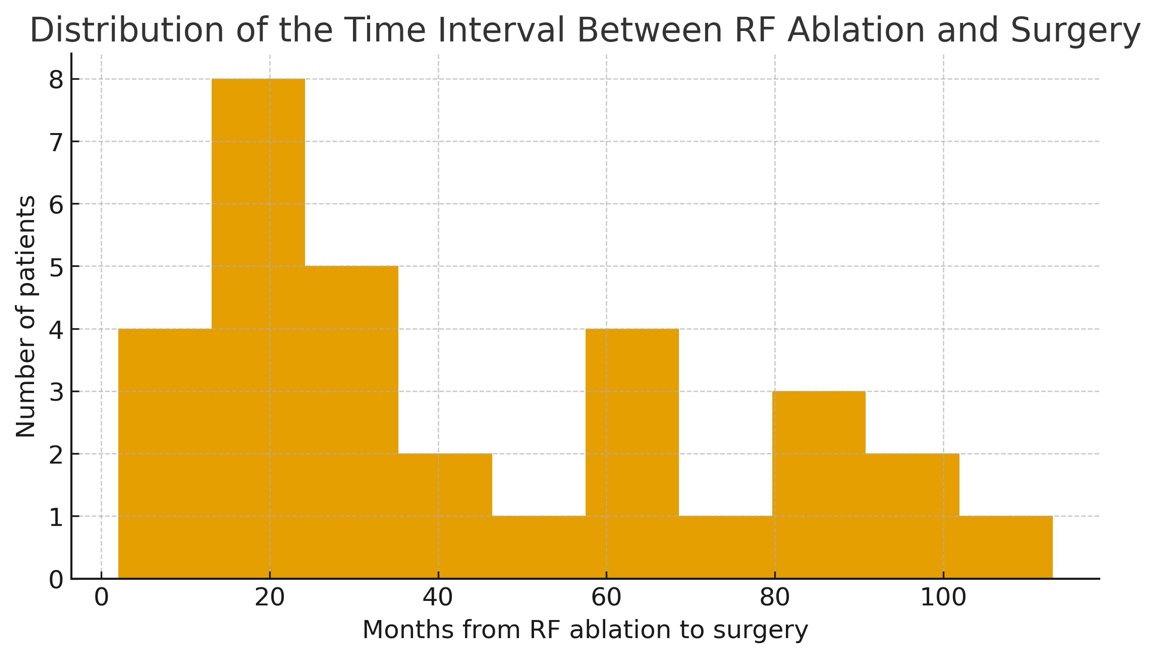


Figure 5s: Boxplot showing the distribution of the ablative response. The plot illustrates the variability in percentage volume change after ablation, including the median, interquartile range, and dispersion of individual data points.


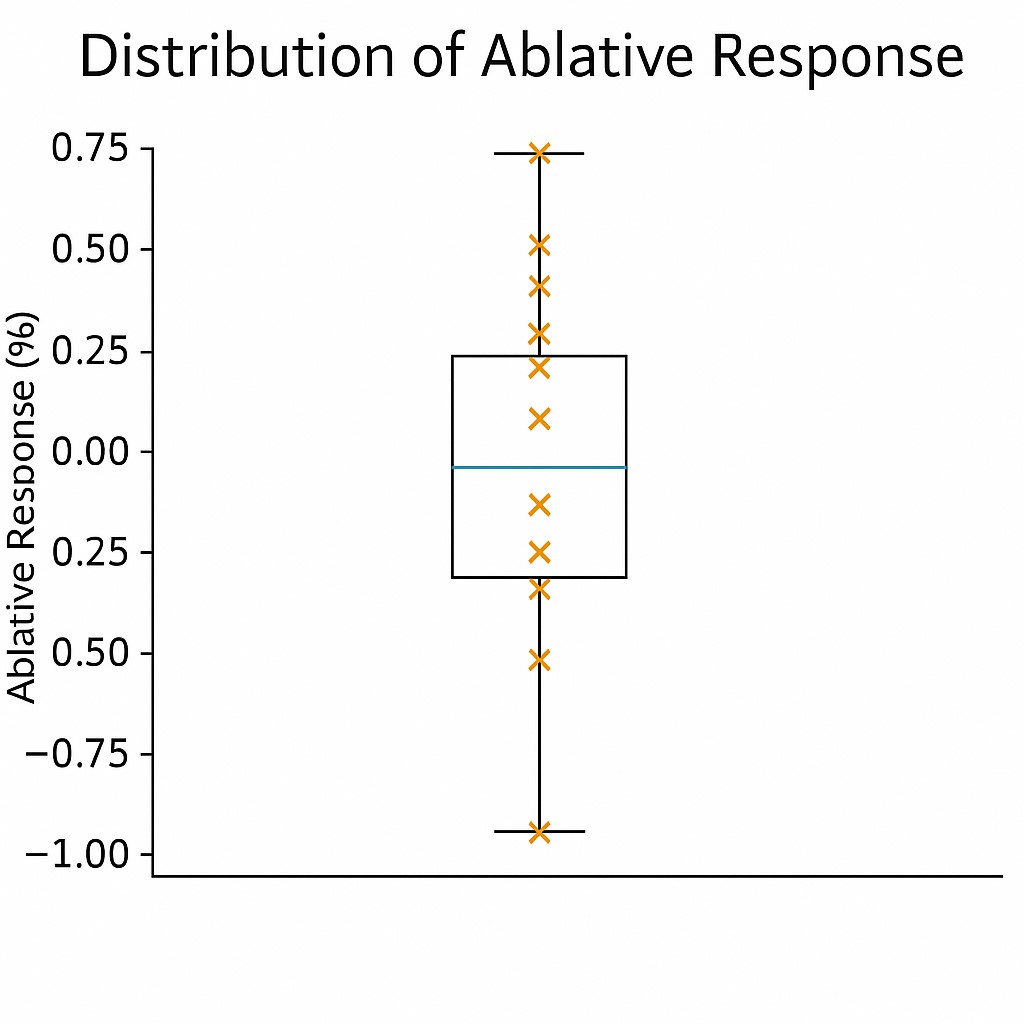


| Table 1s. Nodule characteristics and ablative treatment details at the time of radiofrequency ablation (n. 31). The table presents nodule size at the time of ablation, the number and type of ablative sessions performed, and the percentage volume reduction (ablative response) achieved. Values are given as counts, percentages, medians with interquartile ranges, or means with standard deviations, as appropriate. | | | | | |
| --- | --- | --- | --- | --- | --- |
| Variable | n | % | Median | IQR | Mean ± SD |
| Nodule size at ablation | - | - | 45 mm | 41–50 | - |
| NUMBER OF PROCEDURES |  |  |  |  |  |
| 1 procedure | 26 | 83,9 | - | - | - |
| 2 procedures | 5 | 16,1 | - | - | - |
|  |  |  |  |  |  |
| TYPE OF ABLATION |  |  |  |  |  |
| Thermal ablation | 21 | 67,7 | - | - | - |
| Ethanol ablation | 9 | 29,0 | - | - | - |
| Combined | 1 | 3,2 | - | - | - |
|  |  |  |  |  |  |
| Ablative response (%) | 18 | 58,1 | -0,15 | (-0,43 – +0,16) | -0,16 ± 0,46 |

**Table 2s-a**. Quantitative histopathological composition of post-ablation thyroid nodules. The table presents the proportions of sclerosis, coagulative necrosis, and residual cellular viability within the treated nodules, expressed as the mean, median, range, and standard deviation of the percentage area involved.

| Variable | Mean (%) | Median (%) | Range (%) | Standard deviation |
| --- | --- | --- | --- | --- |
| Sclerosis | 19,2 | 20 | 5–45 | 10,6 |
| Coagulative necrosis | 14,6 | 5 | 0–80 | 20,3 |
| Residual cellular viability | 66,1 | 70 | 5–95 | 24,3 |

**Table 2s-b**. Qualitative histopathological features and associated findings in post-ablation thyroid nodules. The table summarises capsule characteristics, nodule–parenchyma interface, nodule–capsule distance, thyroid and perithyroid soft tissue alterations, and the presence of concomitant carcinoma, reported as the distribution of cases within each category

| Variable | Main category | Distribution (%) |
| --- | --- | --- |
| Nodule capsule | Thin / thick | 77,4 / 16,1 |
| Nodule–parenchyma interface | Regular / chronic inflammation | 61,3 / 19,4 |
| Nodule–capsule distance | <1 mm | 77,0 |
| Thyroid capsule alterations | Absent | 93,5 |
| Perithyroid soft-tissue alterations | Absent / inflammation or hemorrhage | 45,2 / 45,0 |
| Concomitant carcinoma | Absent / present | 87,1 / 6,4 |
